# Accurate dried blood spots collection in the community using non-medically trained personnel could support scaling up routine viral load testing in resource limited settings

**DOI:** 10.1371/journal.pone.0223573

**Published:** 2019-10-17

**Authors:** Kombatende Sikombe, Cardinal Hantuba, Kalo Musukuma, Anjali Sharma, Nancy Padian, Charles Holmes, Nancy Czaicki, Sandra Simbeza, Paul Somwe, Carolyn Bolton-Moore, Izukanji Sikazwe, Elvin Geng

**Affiliations:** 1 Centre for Infectious Diseases Research in Zambia, Lusaka, Zambia; 2 Division of Epidemiology, University of California, Berkeley, Berkeley, California, United States of America; 3 Division of Infectious Diseases, Johns Hopkins University School of Medicine, Baltimore, Maryland, United States of America; 4 Center for Global Health and Quality, Georgetown University, Washington, District of Columbia, United States of America; 5 Division of HIV, Infectious Diseases and Global Medicine, University of California, San Francisco, Zuckerberg San Francisco General Hospital, San Francisco, California, United States of America; 6 Division of Infectious Diseases, University of Alabama, Birmingham, Alabama, United States of America; CIRCB - Chantal BIYA International Reference Centre for research on HIV/AIDS prevention and management, CAMEROON

## Abstract

Regular plasma HIV-RNA testing for persons living with HIV on antiretroviral therapy (ART) is now the global standard, but as many as 60% of persons in Africa today on ART do not have access to standard laboratory HIV-RNA assays. As a result, patients in Zambia often receive treatment without any means of determining true virologic failure, which poses a risk of premature switch of ART regimens and widespread HIV drug resistance. Dry blood spots (DBS) on the other hand require unskilled personnel and less complex storage supply chain so are ideal to capture viral-load results from HIV patients outside clinic settings. We assess collection of DBS in the community using non-medically trained personnel (NMP) and documented challenges. We trained 23 NMP to collect DBS from lost to follow-up (LTFU) patients in 4 rural and urban Zambian districts. We developed a phlebotomy box to transport DBS without contamination at ambient temperature and concomitant training and standard operating procedures. We evaluated this through field observations, bi-weekly meetings, reports, and staff meetings. The laboratory assessed DBS quality for testing validity. We attempted to collect DBS from 357 participants in the community. Though individual reasons for refusal from the remaining 37% were not collected, NMPs reported privacy concerns, awkward box-size which drew attention in the community and fears of undisclosed uses of samples related to witchcraft and circulating narratives about past research. Successful DBS collection was not associated with patient gender, age, time on ART, enrolment CD4, facility. DBS viral-load collection by NMP is feasible in Zambia. Our training approach and assessments of NMP not part of the health system can be extended to patients by giving them more responsibility to manage their own differentiated care groups. Concerted efforts that compare collection of DBS by NMP to those collected by skilled-medical personnel are needed.

## Introduction

Although there is consensus that HIV RNA monitoring is the preferred approach to monitoring HIV treatment, [1–8] access to the expensive and technically demanding test remains limited in sub-Saharan Africa [6,9–12]. It is estimated that only 60% of all people living with HIV in Africa have access to standard plasma HIV RNA testing, leading to substantial risks of unmeasured virologic failure from HIV treatment, and HIV drug resistance (HIVDR) in the region [10,11,13,14]. Collection of blood plasma through venepuncture, required for standard VL monitoring is particularly difficult in decentralized settings due to training, transport costs to laboratory facilities, and complexity in shipping and storage requirements [4,15]. Access to VL monitoring is difficult though there are 450 laboratories across the 10 provinces in Zambia, however capacity to conduct VL assays is limited to provincial level hospitals only (1 per province). Zambia’s 2017 adoption of treatment guidelines in which all people living with HIV are eligible for treatment, combined with the UNAIDS goal of 90% viral suppression, has further increased demand for viral load testing. Furthermore, care in Zambia is increasingly being delivered outside of clinics and in the community which introduces further challenges for traditional plasma viral load testing [16].

Dried blood spots (DBS) on the other hand, can overcome some of these requirements and help make HIV RNA monitoring more widely accessible. Although as compared to standard plasma HIV RNA quantification, DBS-based results are less sensitive or specific to viremia. The consequence of misclassification can be ameliorated with different cut-points, and DBS remains a viable means of monitoring treatment success when standard assays cannot be carried out. The public health community needs more information, however, about the promising operational characteristics to understand how best to incorporate this technology into decentralized health systems. With DBS, theoretically, trained NMP can collect the sample in a patient’s comfort obviating the need to travel to a facility for phlebotomy. [11,17]. Most of the data about DBS to date, is focused on laboratory performance characteristics (e.g., sensitivity and specificity) and DBS have already been shown to be a viable alternative to blood plasma venepuncture for Polymerase Chain Reaction (PCR) in early infant diagnosis of HIV-1 infection [18,19].

In order to close this knowledge gap about implementation, we sought to evaluate the ability of NMP in collecting DBS from HIV infected persons in Zambia under routine health service delivery conditions. We sought to assess the implementability of DBS collection in the field using a minimally invasive collection method that could be consistently and easily implemented in the community using unobtrusive NMP. We assessed our DBS collection training for NMP, the feasibility and acceptability among beneficiaries and among NMP and predictors of successful DBS sample collection by NMP in field conditions.

## Materials and methods

### Study population and setting

Between September 2015 and July 2016, 23 non-medically trained personnel were recruited and trained to collect DBS from HIV-1- infected adults on ART identified as lost to follow up (LTFU) from HIV care, defined as 90 or more days late for their last appointment in Zambia’s electronic medical record (EMR) from 13 health facilities in Lusaka Province [20,21]. Collecting DBS in the field for HIV RNA quantification was carried out as part of a larger parent study [20,21] to estimate the prevalence of viremia after accounting for normally unmeasured LTFU patients. NMP were recruited on the basis of having higher secondary education, excellent communication with interpersonal skills and ability to work confidentially with sensitive information. The 13 clinics received technical support from the Centre for Infectious Disease Research in Zambia (CIDRZ). CIDRZ has since 2004 supported the roll out of PEPFAR-supported National ART services in 4 provinces with over 165,000 individuals on ART having made at least one visit two years before the project [20].

#### Procedures and measurements

NMP were trained to identify eligible participants, obtain consent, collect DBS specimens and process samples (e.g., labelling, transport). We collected participant demographics, clinical history, and ART adherence data. ART history, including date of ART initiation, and WHO clinical stage, was abstracted from patient clinic records.

#### Sample collection procedures and transport

We engaged in a systematic process to develop our approach to community-based DBS collection by conducting multiple interviews and observations of an on-going study within CIDRZ to adapt steps in DBS collection that ensure effective sample storage, transportation and quality. Interviews with facility-based staff, laboratory and fleet personnel to determine inputs needed for effective field DBS collection were conducted. We assessed barriers experienced during blood collection, the facilitators identified, and recommendations for improved service delivery. We conducted web-based searches on guidelines for DBS collection and interacted with other CIDRZ study personnel to understand how they collected DBS from infants in the community. Web based searches included the following terms; ((dried or dry) and blood and spot*), (DB for HIV monitoring), (DBS and viral load and HIV), (finger prick blood sample collection in the community) and (task shifting viral load monitoring to community health workers). Finally, we visited various medical equipment suppliers in Zambia to source suitable lightweight phlebotomy boxes that could be used for field collection. This process informed the design of a simplified transportation and storage system to transfer DBS from the community to the laboratory and facility. Capillary blood was collected on Whatman grade 903 filter paper by NMP who had completed practical and didactic training per study protocol. After collection, we air-dried DBS specimens through placing them in a transportable drying rack in the field where specimens were pinned to Styrofoam in our phlebotomy box to retain a horizontal position that did not touch other surfaces. DBS were transported to the nearest health facility using a study motorbike fitted with a carrier to keep the phlebotomy box used for DBS collection and transportation upright at all times. Once at the facility, the DBS card was air dried for a further 4 hours (though preferably overnight) prior to being placed in a zip lock plastic bag with a desiccant and transported to the laboratory within 14 days of collection using a routine carrier.

To assess implementation, we captured information about completion of selected steps in this process. DBS field collection competency was assessed by the study Quality Assurance and Quality Control team (QA-QC) using a 14-question checklist (S1 Appendix). This tool identified key areas for booster training. Booster training: (1) reinforced specific aspects of in-person training and (2) provided additional training on new issues encountered in the field. Our question checklist focused on assessments of sample collection procedures (ensuring a participant’s hands were warmed, use of alcohol swabs, correct use of requisition form, disposal of sharps, packaging of DBS), sample quality, patient engagement and consent in the study. It was developed using available online resources and the topics covered in the DBS training package. Each NMP was observed at least once during the study period.

#### Didactic and practical training

We used competency-based training for NMP and in-service booster training. Training on DBS collection was provided by an experienced trainer and included a half day didactic presentation and another half day demonstration of DBS collection and transportation from community to central laboratory. Additional training included a half-day skills development/ practice session for small classes of four NMP, making observations of competency in specimen collection. Results from observations of role plays of DBS collection and a written in class assessment on principles of correct DBS collection were used to provide targeted individual training. The class competency assessment tool is presented in supplementary materials (S2 Appendix). NMP were trained to air dry DBS for 4 hours (though preferably overnight) prior to being placed in a zip lock plastic bag and transported at ambient temperature to the CIDRZ central laboratory in Lusaka within 14 days of collection using a routine transport courier (S3 Appendix). NMPs assessed the samples for quality to ensure that blood spots 1) filled the predetermined circle on the filter paper; 2) adequately soaked through to have similar size on both sides of the filter paper to rule out smearing from finger contact on the collection paper; and, 3) did not overlap with one another. DBS samples were collected between October 2015 and May 2016.

#### DBS sample quality and challenges

The COBAS^®^ AmpliPrep/COBAS^®^ TaqMan^®^ HIV-1 Test, v2.0 was used for the quantification of Human Immunodeficiency Virus Type 1 (HIV-1) RNA in DBS. The results from the VL DBS testing are reported by Sikazwe et al. where sensitivity and specificity to detect treatment failure (threshold of 1000 copies/mL) from DBS were determined to be 80.8% and 87.3% respectively [21]. Trained laboratory staff provided qualitative feedback on specimens collected on a bi weekly basis. DBS quality was assessed by examining the adequacy of drying before being packaged to the laboratory, absence of clotting, sufficient blood spot and accurate labelling of sample as per laboratory standards. We conducted bi-weekly staff meetings with NMP and discussed the challenges faced in the DBS collection component of the study such as community/ participant reactions to DBS collection. Four Focus Group Discussions (FGDs) of 5–6 NMP purposively selected for their role in DBS collection were also conducted once during study implementation and then at the end of the study. Discussions notes were taken for transcription and thematic analysis. The FGD question guide was developed in response to the experiences reported from individuals during study implementation. In each FGD, we discussed challenges during DBS collection in the field, both practical and socio-cultural.

### Analysis

FGDs were not audio recorded hence detailed field notes were used as part of our summary process, to articulate our interpretations of the data from the FGDs. Our analysis sought relationships between various themes that were identified during discussions with the NMP by constant comparative analysis. Descriptive statistics on the competency test and field evaluation performance were used to summarize NMP understanding of processes. Bivariate and multilevel mixed effects logistic regression models were used to assess factors associated with successful DBS collection (suitable for testing by the laboratory). Sociodemographic information on samples was collected from the Laboratory Information Management System (LIMS) and EMR. Sex, age, years on ART, and evidence of CD4 count and facility type were used in the model.

### Ethics

Ethical approval was obtained from the University of Zambia Biomedical Research Ethics Committee (UNZABREC), University of Alabama at Birmingham (UAB) institutional review boards (IRB) and Ministry of Health (MOH) prior to initiating study procedures. All participants provided written informed consent which was recorded on the consent form. All IRBs approved this consent procedure.

## Results

### Blood collection

**Fig 1:**
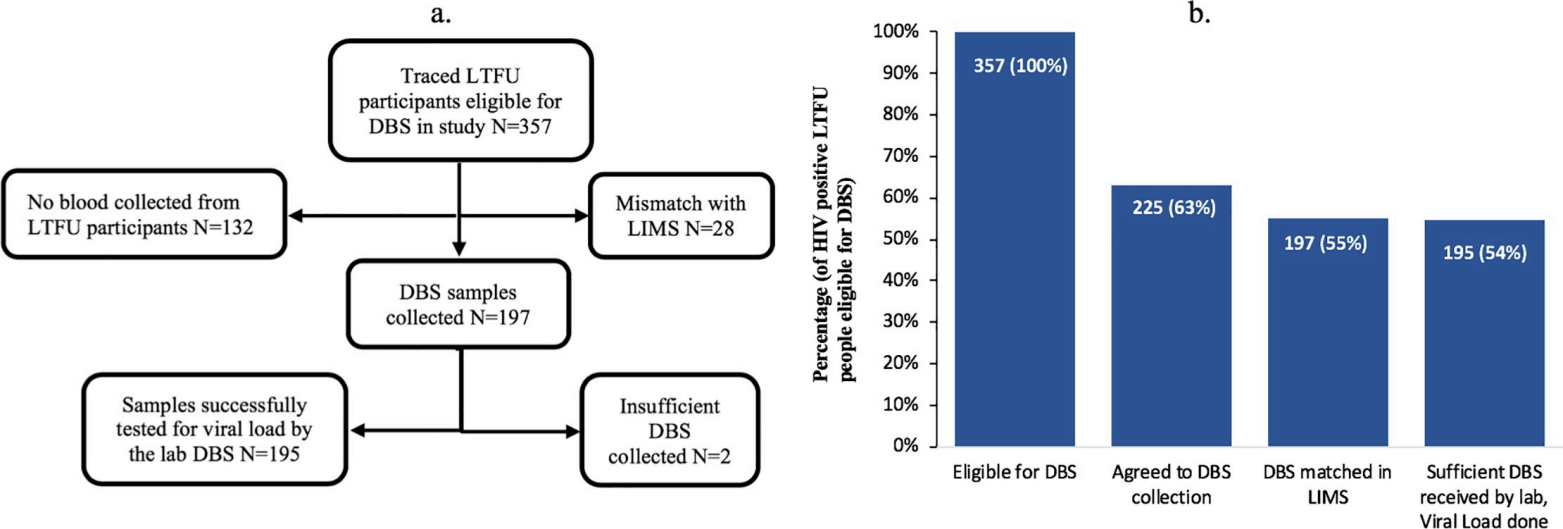
DBS study population and collection cascade. (A) Flow diagram of enrolment and DBS follow-up (B) DBS collection cascade. LTFU, lost to follow-up; DBS, Dried Blood Spot; LIMS, Laboratory Information Management System. Data shown is from 13 study sites.

The parent study identified 357 LTFU individuals from EMR as eligible for DBS to assess virologic suppression. Just over 40% were male, median age of 36 (IQR: 30–41) with a median enrolment CD4 count of 248 cells/μL (IQR: 131–391). Demographic and clinical characteristics of this population are described in Table 1. We successfully determined the whereabouts of all 357 LTFU individuals for whom blood draw was successful for 197 participants (Fig 1A). No blood was collected from 132 (37%). Of those, the vast majority or 123 (93%) refused blood draws, 7 (1.9%) were out of town or dead, and 1 (0.3%) had fingers too callused for DBS collection. Fig 1B shows that patient engagement had the most influence for successful DBS collection. Our bivariate and multivariate logistic regression model for predictors of successful DBS collection did not yield any significant associations with patient gender, age, time on ART, enrolment CD4 nor facility type.

**Table 1:** Characteristics of all patients eligible for DBS between 19 October 2016 and 31 May 2017, N = 357.

BASELINE CHARACTERISTICS	N = 357
Male Sex, n (%)	153 (41)
Median Age, years (IQR)	36 (30–41)
Time on ART, years (IQR)	1.3 (0.33–3.53)
Enrolment CD4 count, cells/μL (IQR)	248 (131–391)
**WHO clinic stage at initiation, n (%)**	
Stage 1	166 (49)
Stage 2	55 (16)
Stage 3	105 (31)
Stage 4	12 (2.6)
Prior History of TB, n (%)	238 (63.6)
**Marital Status, n (%)**	
Single	64 (18)
Married	233 (64)
Divorced	36 (10)
Widowed	31 (9)
**Highest Education, n (%)**	
None	9 (2.5)
Lower mid-basic	129 (35)
Upper basic/secondary	166 (45)
College/ University	61 (17)
**Facility type, n (%)**	
Hospital	45 (12)
Rural	38 (10)
Urban	291 (78)

Values are N (%) or median, interquartile range (IQR). ART, Antiretroviral therapy; WHO, World Health Organization; TB, Tuberculosis

### Competency of non-medical personnel

**Fig 2:**
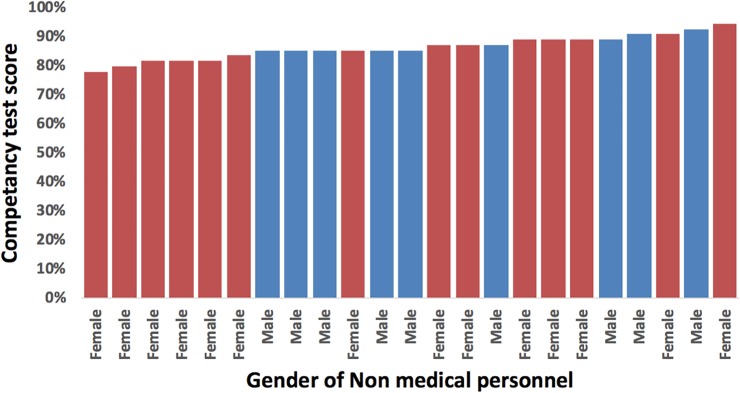
Competency test scores for trained non-medical personnel. Test scores assessed understanding of materials required for dried blood spot (DBS) collection, collection procedures and air drying for finger prick DBS, packaging and transportation of DBS and response to needle stick injury.

All 23 NMP passed and completed the competency test with an average score of 86% (Fig 2.). Competency test scores were calculated as a percentage of correct responses out of total questions asked. NMP were evaluated in the field at least once and combined with laboratory reports of insufficient or poor-quality samples, 9 received targeted booster training on topics ranging from collection and drying of DBS, packaging of DBS and procedure for needle stick injury. Common problems discussed during bi-weekly feedback meetings included challenges in sample collection based on competency results, privacy during blood collection and participant acceptance of blood collection.

### Laboratory assessment

Fig 1 shows that 28 (8%) of the 357 eligible for DBS were rejected by the laboratory due to inappropriate labelling (mismatch between the patient ID from the facility EMR and LIMS). In total, 197 DBS samples, were successfully collected for viral load testing. The laboratory successfully tested 98% (N = 195) of DBS samples. 1% (N = 2) of the 197 contained insufficient blood when assessed by the laboratory and were therefore rejected. Detailed results on how we managed bias incurred via the inaccuracy of DBS-based viral load results by using the documented sensitivity of 80.8% and specificity of 87.3% (for detecting a viral load of ≥ 1,000 copies/ml) as compared to plasma HIV RNA determination through an outcome misclassification correction approach are provided in Sikazwe et al [21].

### Qualitative findings of DBS collection

Table 2 shows the sample DBS questions for FGDs. Overall, NMP reported enthusiasm for DBS collection among patients in bi-weekly meetings and the FGDs conducted at the beginning and end of tracing. In the four FGDs with 23 NMP, a high frequency revealed that participants wanted more control over their health and were interested in the DBS collection process, saying that *“In the field*, *patients want* [VL] results [from DBS collection] *then and there*.*”* However, NMP also discussed environmental and social constraints to patient privacy and confidentiality.

**Table 2:** Sample questions for Focus Group Discussions with NMPs.

	Question
1)	Describe your experience of collecting DBS in the community
2)	Describe the response from the community
3)	Why do patients accept DBS collection
4)	What are the main reasons for a patient refusing DBS collection
5)	Do patients understand the importance of Viral Load

NMP, non-medical personnel; DBS, dried blood spots

#### Lack of privacy

At the first meeting, it was often difficult to find a private place where nobody could witness the process of finger prick and blood spotting such that “s*ometimes you have to assess a patients’ home and maybe come back*” and that “*Some patients opt for clinic-based DBS collection*”.

NMP were unable to freely interview and collect DBS samples for some participants because of home environments that lacked privacy from other family members. Due to stigma, it is common for HIV-infected LTFU participants to not disclose their status to other family members or work colleagues[22,23]; hence appointments were made to meet with participants willing to take part in the study at a time or place when privacy was assured (Table 3).

**Table 3:** Qualitative results from DBS collection experience.

Theme	Quote
*Privacy*	*“We had to make appointments because when we went with the box*, *people would run away…we needed a smaller DBS box”*
	*“The issue with DBS is privacy*. *People were suspicious even of rucksacks”*
	*“The size of the bag triggers suspicion*. *A change in bags is necessary…”*
*Stigma and Confidentiality*	*“They have a problem with the staff*. *Sometimes Health Care Workers (HCW) expose patients in the community…very common for that clinic”*

DBS, dried blood spots

Our NMP initially traced patients without the phlebotomy box so as to protect the privacy of individuals due to suspicion it raises among other community members. Once they identified the patient and explained the process, they returned with their phlebotomy box to collect the DBS sample, which was not always well received (Table 3).

#### Lack of confidentiality and associated stigma

Even if privacy was assured regardless of wanting to collect DBS, there was no guarantee that interviews would not be overheard within the home. NMP assessed the physical environment where they met with the participant and in most cases, would follow the participant’s lead on whether a particular environment was suitable. If the participant determined it unsuitable, the NMP asked for suitable alternatives.

Study NMP found that participants had disclosure concerns due to poor experiences with health workers telling other community members about their HIV status [22–24]. According to our study NMP, this affected the welcome NMP received in communities where they were perceived to be health care workers.

Study NMP also described how clinic staff contribute to stigma in the community by not maintaining confidentiality and disclosing who is living with HIV. NMP reported multiple issues related to disclosure the can arise in the community setting, including fear of direct disclosure by staff and fear of disclosure by association with the clinic (Table 3). Fear of rumours starting about the community-based blood collection also affected recruitment of study participants, and the relationship between the NMP/researchers and the study sample. They described stigma by way of disclosure concerns and public attitude as the main reason for refusing blood collection, fearing for example that people *“would say why that house …”*. The study addressed this by ensuring staff did not wear identifiable clothes and strictly adhering to agreed appointments for blood collection.

## Discussion

Our findings suggest that it is feasible to train and support NMP to competently collect finger prick DBS samples for viral load testing under field conditions and in areas which cannot be served by standard laboratory networks in sub-Saharan Africa [11,17,25,26]. The provision of free ART services regardless of CD4 count in Zambia has created unprecedented health systems demand that cannot be met using traditional viral load monitoring techniques that require a lot of infrastructure and utilization of highly skilled personnel [25,27,28]. With all but 2 samples viable for VL analysis and over 90% clearly linked to specific individuals, we have successfully shown that DBS collection by NMP allows us to monitor VL collected in the community. The mismatching of 28 samples was due to the failure of the healthcare system in maintaining unique patient identifiers in the EMR at the facility and LIMS [29].

Overall, this paper provides a detailed description of the operational challenges associated with collecting DBS in the field and suggest that this approach should be incorporated in settings where plasma viral load monitoring is not feasible, including in treatment models that are predominantly or mostly community based. We found the largest drop off to be related to patient refusal. While we did not collect reasons for refusal, the population which we sought were patients who had been LTFU, who have a high prevalence of stigma and other barriers to treatment[30,31]. In contrast, if the patients agreed, very few failures at the collection process, transport, labelling, nor laboratory testing would have occurred. Indeed, the operational challenges are mostly patient-based, whereas the technical dimensions occurred smoothly. In short, where health systems need to collect DBS in the field, patient engagement had the greatest influence on success. Our results show that NMPs can be used to improve access to VL monitoring in remote settings. A detailed understanding of how the contextual challenges faced not only by NMPs but also patients in the community can help strengthen DBS VL monitoring. However there is a broad consensus about the ability to enhance delivery of basic services to child care-takers (maternal, child and women’s health [MCH] interventions) and ART patients using community health workers therefore leading to improvements in mortality [32]. It is true that these improvements are not the case in all settings and implementing programmes using NMP has been a challenge but the benefits still outweigh the disadvantages. In our setting, non clinical personal are often associated with HIV care follow up, for example LTFU tracing [20,21]. As this was a research study that encouraged patient confidentiality, it is likely that uptake of DBS VL in the community can be increased through sensitization through key health promotional messages in larger public health programmes and also by receiving supervisory support from professional health care workers at the facility to ensure quality by alignment to the broader health system.

With an average competency score of 86%, our ability to competently train NMP has the potential to expand differentiated services without overly straining finances by task shifting highly skilled processes to less skilled personnel as has been done in Zambia and others by use of lay health care workers in response to the human resource demands of HIV care and treatment [27,33,34]. NMP were recruited from populations similar to people living with HIV (PLHIV) i.e. non-medically trained, meaning that it may be possible to empower patients to collect DBS VL samples within their own differentiated community adherence groups. This would further reduce the need for routine clinic/ laboratory visits which are costly and overburden both patients and facilities, releasing staff to pay closer attention to patients with unsuppressed VL [16,27,35]. This represents an important element of health systems strengthening, especially insofar as many countries in Sub-Saharan Africa (SSA) are implementing test and treat all and VL testing without sufficient and sophisticated equipment and skilled personnel [1,28,36,37], suggesting that VL implementation currently falls well short of guidelines and limits the public health impact of programmes.

Although NMPs reported a desire by patients to have blood drawn by DBS, disregard for patient confidentiality and professionalism by health care workers who work and reside in the same community as participants must be addressed to regain public trust [23]. The collection of DBS by NMPs in the community was potentially hindered by the poor patient experience with health care workers at the facility and in the community, resulting in patients disengaging from healthcare services [31,38–41]. Based on the findings from our study staff, measures to protect patient confidentiality such as the use of much smaller boxes concealed in a gym bag and identifying community spaces such as schools and churches where privacy and confidentiality can be maintained could increase uptake. Adopting a three-pronged approach of training, mentorship, and continuous quality assessment could allow for the rapid roll-out of VL monitoring by NMP in resource limited settings.

### Conclusions

This is the first study to describe the processes of developing and implementing DBS collection by non-medically trained personnel in field conditions. Use of NMP can serve as an important catalyst in promoting adoption of VL, ensuring better patient outcomes, and enabling vulnerable patient groups and those in rural areas to be reached more effectively. The collection of DBS also shows promise for field-based collection including by HIV patients themselves in differentiated service delivery models, which would reduce travel costs for patients, decongest facilities and for national HIVDR surveillance. Identification of community spaces to ensure privacy and confidentiality along with structured programmes for stigma reduction could increase participation in community-based DBS collection.

### Limitations

Our study should be considered with the following limitations. Just over a third of our sample (37%) refused DBS collection, and our NMPs uncovered fears of lack of privacy and confidentiality, perceived stigma, and dislike of unnecessary procedures, all of which can be addressed albeit with considerable effort to minimize the size of the box (S3 Appendix) [42,43]. Of note, the study population is one that previously disengaged from HIV care provided by medically trained personell. We believe the refusal rate observed in this study is indicative of perceived benefit and trustworthiness in interactions between systems, individuals and patients. We recruited NMP who were familiar with the communities in which the facilities were based. Successful DBS collection by NMP relies on the elevated level of group participation and beneficial links between NMP and the health system. Because of this, the role that trust plays in the work on NMP could have been underestimated [32]. Despite these challenges, we believe there are important lessons that come out of our experience collecting DBS in the community.

Our findings are based on a review of implementation documents and rapid data collection by QA-QC and study managers to make mid-course corrections in procedures. Careful consideration will need to be given to ensuring quality control and performing cost-effectiveness analysis on costs associated with training and supporting DBS collection. This study was based on a cohort of LTFU patients who behave differently to patients engaged in HIV care. Our FGDs were not audio recorded, however detailed notes were taken.

### Implications

The ease of collecting and transporting DBS is invaluable given that current laboratory infrastructure and the relative complexity of techniques preclude decentralization of VL and HIVDR testing beyond second tier referral laboratories. DBS collection by NMP is potentially suitable to use in community-based settings. A WHO survey in low and middle-income countries found that only 60% of patients on ART receive VL testing with the demand for VL testing in Africa having reached 9.5 million in 2018. Task shifting to NMP to collect DBS would mean patients on ART could be monitored regularly for VL and HIVDR so that their ART regimen can be modified according to their resistance profile. DBS collection that is logistically feasible, cost saving and responsive to patient demand, therefore has a critical role in health systems innovating to attain the 90-90-90 targets [1,8,17]. The ability to collect DBS in non-clinical settings makes it a realistic routine component of care, especially in differentiated service delivery settings where patients might meet in schools and other community settings and receive services such as adherence counselling and ART drug delivery [44].

## Supporting information

S1 Appendix 14-question field checklist. Questions used to assess field implementation of dried blood spot collection by non-medical personnel.
(PDF)

S2 Appendix Competency assessment tool. Assessment tool used to assess knowledge of dried blood spot (DBS) collection procedures by non-medical personnel post didactic and practical training.
(PDF)

S3 Appendix DBS collection box with partitioning. Modified first aid box partitioned with Styrofoam to hold dired blood spots (DBS) horizontally at all times during transportation.
(TIFF)

## References

[1] World Health Organization. Guidelines Guideline on When To Start Antiretroviral Therapy and on Pre-Exposure Prophylaxis for Hiv World Health Organization 2015; 78. 978 92 4 150956 5 26598776

[2] Quinn TC, Wawer MJ, Sewankambo N, Serwadda D, Li C, Wabwire-Mangen F, et al Viral Load and Heterosexual Transmission of Human Immunodeficiency Virus Type 1. New England Journal of Medicine. 2000;342: 921–929. 10.1056/NEJM200003303421303 10738050

[3] Monleau M, Aghokeng A. Field evaluation of dried blood spots for routine HIV-1 viral load and drug resistance monitoring in patients receiving antiretroviral therapy in Africa and Asia. Journal of clinical. 2014; 10.1128/JCM.02860-13 PMC3911301 24478491

[4] Hamers R, Kityo C, Lange J, Wit T de, Mugyenyi P. Global threat from drug resistant HIV in sub-Saharan Africa. Bmj. 2012; 10.1136/bmj.e4159 22709963

[5] Bennett DE, Bertagnolio S, Sutherland D, Gilks CF. The World Health Organization’s global strategy for prevention and assessment of HIV drug resistance Antiretroviral treatment scale-up in resource- limited countries. 18578063

[6] Bertagnolio S, Parkin NT, Jordan M, Brooks J, Gerardo García-Lerma J. Silvia Bertagnolio, et al: Dried Blood Spots for HIV-1 Drug Resistance and Viral Load Testing Dried blood spots for HIV-1 Drug Resistance and Viral Load Testing: A Review of Current Knowledge and WHO Efforts for Global HIV Drug Resistance Surveillance. 2010; 21179184

[7] WHO. WHO Manual for HIV Drug Resistance Testing Using Dried Blood Spot Specimens. Who/Hiv/201230. 2012; 29.

[8] UNAIDS. 90-90-90 An ambitious treatment target to help end the AIDS epidemic. http://wwwunaidsorg/Sites/Default/Files/Media_Asset/90-90-90_En_0Pdf.2014; 40.

[9] Ziemniak C, Mengistu Y, Ruff A, Chen Y-H, Khaki L, Bedri A, et al Use of dried-blood-spot samples and in-house assays to identify antiretroviral drug resistance in HIV-infected children in resource-constrained settings. Journal of clinical microbiology. American Society for Microbiology; 2011;49: 4077–82. 10.1128/JCM.01004-11 21956987 PMC3232965

[10] Keiser O, Chi BH, Gsponer T, Boulle A, Orrell C, Phiri S, et al Outcomes of antiretroviral treatment in programmes with and without routine viral load monitoring in Southern Africa. AIDS (London, England). NIH Public Access; 2011;25: 1761–9. 10.1097/QAD.0b013e328349822f 21681057 PMC3605707

[11] Johannessen A, Garrido C, Zahonero N, Sandvik L, Naman E, Kivuyo SL, et al Dried Blood Spots Perform Well in Viral Load Monitoring of Patients Who Receive Antiretroviral Treatment in Rural Tanzania. Clinical Infectious Diseases. WHO, Geneva; 2009;49: 976–981. 10.1086/605502 19663598

[12] Rottinghaus EEK, Ugbena R, Diallo K, Bassey O, Azeez A, DeVos J, et al Dried Blood Spot Specimens Are a Suitable Alternative Sample Type for HIV-1 Viral Load Measurement and Drug Resistance Genotyping in Patients Receiving First-Line Antiretroviral Therapy. Clinical Infectious Diseases. 2012;54: 1187–1195. 10.1093/cid/cis015 22412066 PMC11528918

[13] Sigaloff KCE, Hamers RL, Wallis CL, Kityo C, Siwale M, Ive P, et al Unnecessary Antiretroviral Treatment Switches and Accumulation of HIV Resistance Mutations; Two Arguments for Viral Load Monitoring in Africa. JAIDS Journal of Acquired Immune Deficiency Syndromes. 2011;58: 23–31. 10.1097/QAI.0b013e318227fc34 21694603

[14] World Health Organization. 2010 Revision. Geneva WHO | Antiretroviral therapy for HIV infection in adults and adolescents. WHO. World Health Organization; 2011; 23741771

[15] McDade T, Williams S, Snodgrass J. What a drop can do: dried blood spots as a minimally invasive method for integrating biomarkers into population-based research. Demography. 2007; 10.1353/dem.2007.0038 18232218

[16] Duncombe C, Rosenblum S, Hellmann N, Holmes C, Wilkinson L, Biot M, et al Reframing HIV care: putting people at the centre of antiretroviral delivery. Tropical Medicine & International Health. 2015;20: 430–447. 10.1111/tmi.12460 25583302 PMC4670701

[17] Mavedzenge SN, Davey C, Chirenje T, Mushati P, Mtetwa S, Dirawo J, et al Finger Prick Dried Blood Spots for HIV Viral Load Measurement in Field Conditions in Zimbabwe. PLoS ONE. Public Library of Science; 2015;10 10.1371/journal.pone.0126878 26001044 PMC4441418

[18] Patton JC, Akkers E, Coovadia AH, Meyers TM, Stevens WS, Sherman GG. Evaluation of dried whole blood spots obtained by heel or finger stick as an alternative to venous blood for diagnosis of human immunodeficiency virus type 1 infection in vertically exposed infants in the routine diagnostic laboratory. Clinical and Vaccine Immunology. 2007;14: 201–203. 10.1128/CVI.00223-06 17167036 PMC1797794

[19] Sherman GGG, Stevens G, Jones SSA, Horsfield P, Stevens WS. Dried blood spots improve access to HIV diagnosis and care for infants in low-resource settings. JAIDS Journal of. 2005;38: 615–617. 10.1097/01.qai.0000143604.71857.5d 15793374

[20] Holmes CB, Sikazwe I, Sikombe K, Eshun-Wilson I, Czaicki N, Beres LK, et al Estimated mortality on HIV treatment among active patients and patients lost to follow-up in 4 provinces of Zambia: Findings from a multistage sampling-based survey. Rosen S, editor. PLOS Medicine. Public Library of Science; 2018;15: e1002489 10.1371/journal.pmed.1002489 29329301 PMC5766235

[21] Sikazwe I, Eshun-Wilson I, Sikombe K, Czaicki N, Somwe P, Mody A, et al Retention and viral suppression in a cohort of HIV patients on antiretroviral therapy in Zambia: Regionally representative estimates using a multistage-sampling-based approach. PLOS Medicine. Public Library of Science; 2019;16: 1–17. 10.1371/journal.pmed.1002811 31150380 PMC6544202

[22] Wolf HT, Halpern-Felsher BL, Bukusi EA, Agot KE, Cohen CR, Auerswald CL. ‘It is all about the fear of being discriminated [against]⋯the person suffering from HIV will not be accepted’: A qualitative study exploring the reasons for loss to follow-up among HIV-positive youth in Kisumu, Kenya. BMC Public Health. BioMed Central; 2014;14: 1154 10.1186/1471-2458-14-1154 25377362 PMC4232620

[23] Topp SM, Mwamba C, Sharma A, Mukamba N, Beres LK, Geng E, et al Rethinking retention: Mapping interactions between multiple factors that influence long-term engagement in HIV care. PLOS ONE. Public Library of Science; 2018;13: e0193641 10.1371/journal.pone.0193641 29538443 PMC5851576

[24] Musheke M, Bond V, Merten S. Individual and contextual factors influencing patient attrition from antiretroviral therapy care in an urban community of Lusaka, Zambia. Journal of the International AIDS Society. International AIDS Society; 2012;15 10.7448/IAS.15.3.17366 22713354 PMC3499928

[25] Roberts T, Cohn J, Bonner K, Hargreaves S. Scale-up of Routine Viral Load Testing in Resource-Poor Settings: Current and Future Implementation Challenges: Table 1. Clinical Infectious Diseases. Oxford University Press; 2016;62: 1043–1048. 10.1093/cid/ciw001 26743094 PMC4803106

[26] Rutstein SE, Hosseinipour MC, Kamwendo D, Soko A, Mkandawire M, Biddle AK, et al Dried Blood Spots for Viral Load Monitoring in Malawi: Feasible and Effective. PLOS ONE. Public Library of Science; 2015;10: e0124748. 10.1371/journal.pone.0124748 PMC4405546 25898365

[27] Morris MB, Chapula BT, Chi BH, Mwango A, Chi HF, Mwanza J, et al Use of task-shifting to rapidly scale-up HIV treatment services: experiences from Lusaka, Zambia. BMC Health Services Research. 2009;9: 5 10.1186/1472-6963-9-5 19134202 PMC2628658

[28] World Health Organization. Technical and operational considerations for implementing HIV viral load testing: Interim technical update Who 2014; 28.

[29] Hickey MD, Omollo D, Salmen CR, Mattah B, Blat C, Ouma GB, et al Movement between facilities for HIV care among a mobile population in Kenya: transfer, loss to follow-up, and reengagement. AIDS Care. Taylor & Francis; 2016;28: 1386–1393. 10.1080/09540121.2016.1179253 27145451 PMC5697146

[30] Geng EH, Odeny TA, Lyamuya R, Nakiwogga-Muwanga A, Diero L, Bwana M, et al Retention in Care and Patient-Reported Reasons for Undocumented Transfer or Stopping Care Among HIV-Infected Patients on Antiretroviral Therapy in Eastern Africa: Application of a Sampling-Based Approach. Clinical Infectious Diseases. Oxford University Press; 2016;62: 935–944. 10.1093/cid/civ1004 26679625 PMC4787603

[31] Tweya H, Feldacker C, Estill J, Jahn A, Ng’ambi W, Ben-Smith A, et al Are They Really Lost? “True” Status and Reasons for Treatment Discontinuation among HIV Infected Patients on Antiretroviral Therapy Considered Lost to Follow Up in Urban Malawi. PLOS ONE. Public Library of Science; 2013;8: e75761 10.1371/journal.pone.0075761 24086627 PMC3784425

[32] Grant M, Wilford A, Haskins L, Phakathi S, Mntambo N, Horwood CM. Trust of community health workers influences the acceptance of community-based maternal and child health services. African Journal of Primary Health Care and Family Medicine. 2017; 10.4102/phcfm.v9i1.1281 28582988 PMC5458568

[33] Sanjana P, Torpey K, Schwarzwalder A, Simumba C, Kasonde P, Nyirenda L, et al Task-shifting HIV counselling and testing services in Zambia: The role of lay counsellors. Human Resources for Health. 2009; 10.1186/1478-4491-7-44 19480710 PMC2692981

[34] Kaindjee-Tjituka F, Sawadogo S, Mutandi G, Maher AD, Salomo N, Mbapaha C, et al Task-shifting point-of-care CD4+ testing to lay health workers in HIV care and treatment services in Namibia. African Journal of Laboratory Medicine. 2017; 10.4102/ajlm.v6i1.643 29159139 PMC5684646

[35] Working Group on Modelling of Antiretroviral Therapy Monitoring Strategies in Sub-Saharan Africa A, Phillips A, Shroufi A, Vojnov L, Cohn J, Roberts T, et al Sustainable HIV treatment in Africa through viral-load-informed differentiated care. Nature. NIH Public Access; 2015;528: S68–S76. 10.1038/nature16046 26633768 PMC4932825

[36] UNAIDS. How Aids Changed Everything [Internet]. 2015. Available: http://www.unaids.org/sites/default/files/media_asset/MDG6Report_en.pdf

[37] Raizes E, Hader S, Birx D. Expansion of Viral Load Testing and the Potential Impact on HIV Drug Resistance. The Journal of infectious diseases. Oxford University Press; 2017;216: S805–S807. 10.1093/infdis/jix432 29206999 PMC5853460

[38] Rachlis B, Ochieng D, Geng E, Rotich E, Ochieng V, Maritim B, et al Implementation and operational research: evaluating outcomes of patients lost to follow-up in a large comprehensive care treatment program in western Kenya. Journal of acquired immune deficiency syndromes (1999). 2015;68: e46–55. 10.1097/QAI.0000000000000492 25692336 PMC4348019

[39] Chimbindi N, Bärnighausen T, Newell M-L. Patient satisfaction with HIV and TB treatment in a public programme in rural KwaZulu-Natal: evidence from patient-exit interviews. BMC Health Services Research. 2014;14: 32 10.1186/1472-6963-14-32 24450409 PMC3904687

[40] Dang BN, Westbrook RA, Black WC, Rodriguez-Barradas MC, Giordano TP. Examining the Link between Patient Satisfaction and Adherence to HIV Care: A Structural Equation Model. PLoS ONE. 2013;8 10.1371/journal.pone.0054729 23382948 PMC3559888

[41] Mody A, Sikazwe I, Czaicki NL, Wa Mwanza M, Savory T, Sikombe K, et al Estimating the real-world effects of expanding antiretroviral treatment eligibility: Evidence from a regression discontinuity analysis in Zambia. PLoS Medicine. 2018;15 10.1371/journal.pmed.1002574 29870531 PMC5988277

[42] Boahen O, Owusu-Agyei S, Febir LG, Tawiah C, Tawiah T, Afari S, et al Community perception and beliefs about blood draw for clinical research in Ghana. Transactions of the Royal Society of Tropical Medicine and Hygiene. Oxford University Press; 2013;107: 261–265. 10.1093/trstmh/trt012 23426114

[43] Berger BE, Ferrans CE, Lashley FR. Measuring stigma in people with HIV: Psychometric assessment of the HIV stigma scale. Research in Nursing & Health. Wiley Online Library; 2001;24: 518–529. 10.1002/nur.10011 11746080

[44] Dorward J, Drain PK, Garrett N. Point-of-care viral load testing and differentiated HIV care. The Lancet HIV. 2018 pp. e8–e9. 10.1016/S2352-3018(17)30211-4 29290227 PMC6003416

