# Supplementary material for: Accurate dried blood spots collection in the community using non-medically trained personnel could support scaling up routine viral load testing in resource limited settings
Source: PLoS One. 2019 Oct 17;14(10):e0223573. doi: 10.1371/journal.pone.0223573 (PMC6797100; doi:10.1371/journal.pone.0223573)
Supplement: S1 Appendix — Questions used to assess field implementation of dried blood spot collection by non-medical personnel. (PDF) [file pone.0223573.s001.pdf]

SUPPLEMENTARY 1: 14-QUESTION DBS FIELD CHECKLIST

DBS FIELD EVALUATION

1. Did tracer engage patient in what was happening?
   Yes No

2. Did tracer ensure patient's hands were warmed up?
   Yes No

3. Were gloves worn before touching anything to be used in DBS collection?
   Yes No

4. Were all DBS collection supplies removed and prepared before starting procedure? (***Tick all that applies***)

**DBS Supplies**	**Tick (√)**
Cotton wool	
Plaster	
Lancet	
DBS card	
Sharps boxes	
Alcohol swab	

5. Did tracer fill in patients details on both DBS card and requisition form before collection? (***Tick all that applies***)

**REQUISITION FORM**	**Tick (√)**	**DBS CARD**	**Tick (√)**
Collection date and time		Collection date and time	
PID		PID	
Sex		Sex	
Study/visit		Clinic/Facility name	
Sample storage			
Clinic/Facility name			

6. Was the patient's finger cleaned with an alcohol swab in one direction?
   Yes No

7. Did tracer ensure that the patients hand was below the heart during DBS collection?
   Yes No

8. Was lancet properly positioned? (i.e. not too close to the tip or side of the finger).
   Yes No

9. Was lancet disposed in sharps boxes?
   Yes No

10. Was puncture done correctly?
    Yes No

11. Was patient's finger messaged properly?
    Yes No

12. Was the collected DBS valid?
    Yes No

13. Were details filled in correctly on the requisition forms?
    Yes No

14. Was the collected DBS packaged properly?
    Yes No
