# Supplementary material for: Accurate dried blood spots collection in the community using non-medically trained personnel could support scaling up routine viral load testing in resource limited settings
Source: PLoS One. 2019 Oct 17;14(10):e0223573. doi: 10.1371/journal.pone.0223573 (PMC6797100; doi:10.1371/journal.pone.0223573)
Supplement: S2 Appendix — Assessment tool used to assess knowledge of dried blood spot (DBS) collection procedures by non-medical personnel post didactic and practical training. (PDF) [file pone.0223573.s002.pdf]

SUPPLEMENTARY 2: DBS COMPETENCY ASSESSMENT TOOL

Better Information for Health in Zambia (BetterInfo) Study

DBS ASSESSMENT FORM

This form is used to measure the understanding levels of participants attending the DBS Training Workshop. After the assessment, the questions which were incorrectly answered will become points of training so as to enhance understanding among participants. (*Please answer the following questions by either circling responses or ticking (√) in the appropriate box*)

1.0 Personal Details

Name of Training Participant: ______________________________

Gender: Male (   )   Female (   )

Date:

Venue:

2.0 Background Questions

2.1 What does DBS stand for? (a) Dry Blood Sampling (b) Dry Blood Spot (c) Dry Blood Specimen.

2.2 How will blood be collected for DBS under Better Info Study? (a) Drawing full blood from a patient (b) Finger prick using a needle (c) Minor cut on the skin (d) Finger prick using a lancet

3.0 Key materials required for DBS

There are key materials required for conducting DBS. Provide your answers by ticking 'true' or 'false' in the spaces provided:

	Key materials required for DBS	True	False
**3.1**	Lasec DBS Card		
**3.2**	No Gas impermeable storage bags		
**3.3**	No Desiccant packs		
**3.4**	Humidity indicator (cards)		
**3.5**	Alcohol swabs		
**3.6**	Talk Time for Phone Calls		
**3.7**	No Bandage/Plaster		
**3.8**	Lancet		
**3.9**	Drying racks		
**3.10**	DBS lab requisition form		
**3.11**	Gloves		
**3.12**	DBS transport and storage box		
**3.13**	Pain Killers		

4.0 Collection of Blood Specimen

Prior to the specimen collection, the DBS cards should be labeled with the patient's details.

4.1 What are the patients' details that should be indicated on the DBS cards? (Select the appropriate answers) (a) Patient name (b) Patient ART number (c) Site name/ Site identification number (e) Blood collection date/time

There are a number of things that should be observed when collecting blood specimen for DBS. Below tick 'true' or 'false' in the spaces provided:

	Process	True	False
**4.2**	Put on any gloves and apply some sanitizer on your hands.		
**4.3**	Handle the DBS card carefully using the edges; NEVER touch the areas where the blood will be collected.		
**4.4**	Use whole blood sample from finger prick.		
**4.5**	Make sure to warm the participant's hand and make sure the hand is below the level of the elbow (to allow gravity to help you collect the blood).		
**4.6**	Select finger. Clean patient finger with disinfectant or alcohol wipe, generally middle or ring finger is preferred, avoid fingers with rings on.		
**4.7**	Allow to air dry for 30 seconds		
**4.8**	Use a sterile, disposable lancet to puncture the skin to the side of the fingertip.		
**4.9**	Dispose of the lancet in the biohazard container		
**4.10**	With the finger extended, wipe away the first drop of blood then allow a large, hanging drop of free-flowing blood to accumulate at the puncture site.		
**4.11**	To collect the drop of blood, touch the filter paper to the edge of the drop, allowing the blood to be drawn into the first circle on the card by capillary action. Allow the finger to touch the card.		
**4.12**	Then, allow another large drop of free-flowing blood to form at the puncture site and collect this drop in the next circle.		
**4.13**	You need multiple drops of blood on top of each other. Continue collecting drops of blood in the same manner until all the circles are filled on the DBS Card.		
**4.14**	If the blood stops flowing before sufficient blood has been collected, gently massage/squeeze the finger to encourage blood droplets formation.		

	Process	True	False
**4.15**	If you are unable to obtain flowing blood using finger stick despite multiple attempts, leave the patient alone and proceed to the next.		
**4.16**	After adequate sample is collected, give the participant gauze or swab to hold pressure to the fingertip. Elevate the fingertip below the elbow.		
**4.17**	After completing the sample collection, place the DBS card on the drying rack. The drying rack should ideally be placed so that the DBS cards dry in the vertical position.		
**4.18**	If no drying rack is available, the DBS card can be laid flat on a clean paper towel at the facility.		
**4.19**	Fill out the study lab requisition form and leave it close to the DBS card, so it can accompany the card to the lab, once dry		
**4.20**	DBS cards must dry for at least 1hour and a heat source can be used sometimes to dry the DBS Cards.		
**4.21**	When dry, the spots will appear a uniform dark brown. The appearance should be similar to that of a dried bloodstain and no areas of red coloration should be seen.		

5.0 Collection and Drying of DBS

There is a standard procedure which should be applied when drying DBS during field tracing. Mark the correct procedure by 'true' or 'false' in the space provided.

	Drying of DBS	True	False
**5.1**	Allow the DBS card to dry sufficiently so blood is not flowing when placed in a transport box		
**5.2**	Appropriately place the DBS card vertically in a rack already provided in the transport box		
**5.3**	The transport box must be carried horizontally at all times.		
**5.4**	When you return to the facility at the end of your tracing activities, ensure that each DBS card has a lab requisition form filled out.		
**5.5**	When you arrive at the facility, remove the DBS card and leave to air dry effectively for 1 hours only.		
**5.6**	Fill out the study lab requisition form. The study lab requisition form is part of record keeping and should be kept by the tracer.		

6.0 Packaging of DBS

6.1 How long should the DBS be kept at room (ambient) temperature? (a) Less than 14 days (b) More than 14 days (c) Both

6.2 What should be done in case genotyping is not performed within the recommended period from the date of collection? (a) Do nothing (b) transport to a central facility (c) transport to a central facility and free to -70°C freeze.

The following are procedures for packaging of DBS. Kindly indicate the correct response by ticking either 'true' or 'false' spaces.

	Packaging of DBS	True	False
**6.3**	Once the DBS card is dry, it should just be put in the first aid kit box.		
**6.4**	Humidity does not cause damage to the HIV virus particles.		
**6.5**	Once completely dry, DBS card should be sealed in its bag from now to when we are ready to test it.		
**6.6**	Bring the plastic bag containing the DBS along with the lab requisition form to the appropriate area so that it can be transported to CIDRZ central laboratory. The requisition form should be stapled to the plastic bag for purposes of identification.		
**6.7**	Insert DBS bag into envelope, Include lab requisitions and specimen delivery checklist, Seal envelope, Label envelope clearly ("BetterInfo DBS specimens") Send to testing lab at Kalingalinga.		

7.0 Procedure for needle sticks injury

7.1 What is a needle stick injury? (a) It is the same as Post Exposure Prophylaxis (b) Injury caused by lancet (c) Injury caused by a needle (d) It is when a/your skin is punctured by a used needle.

7.2 What should happen when a Better Info Staff member suffers a needle stick injury while working with a patient? (a) Immediately wash the site with soap and running water (b) use bleach or other caustic agents to clean the exposed site (c) use numerous amounts of saline or water in case of mucosal exposure (d) use clean water with detol in case of exposure to the eyes (d) contact fellow tracer (e) contact In-Charge/Supervisor/Study Coordinator.

Thank you for taking the time to fill out this DBS assessment form.

Thank You!!!!
