# Supplementary figures and images for: Accurate dried blood spots collection in the community using non-medically trained personnel could support scaling up routine viral load testing in resource limited settings
Source: PLoS One. 2019 Oct 17;14(10):e0223573. doi: 10.1371/journal.pone.0223573 (PMC6797100; doi:10.1371/journal.pone.0223573)

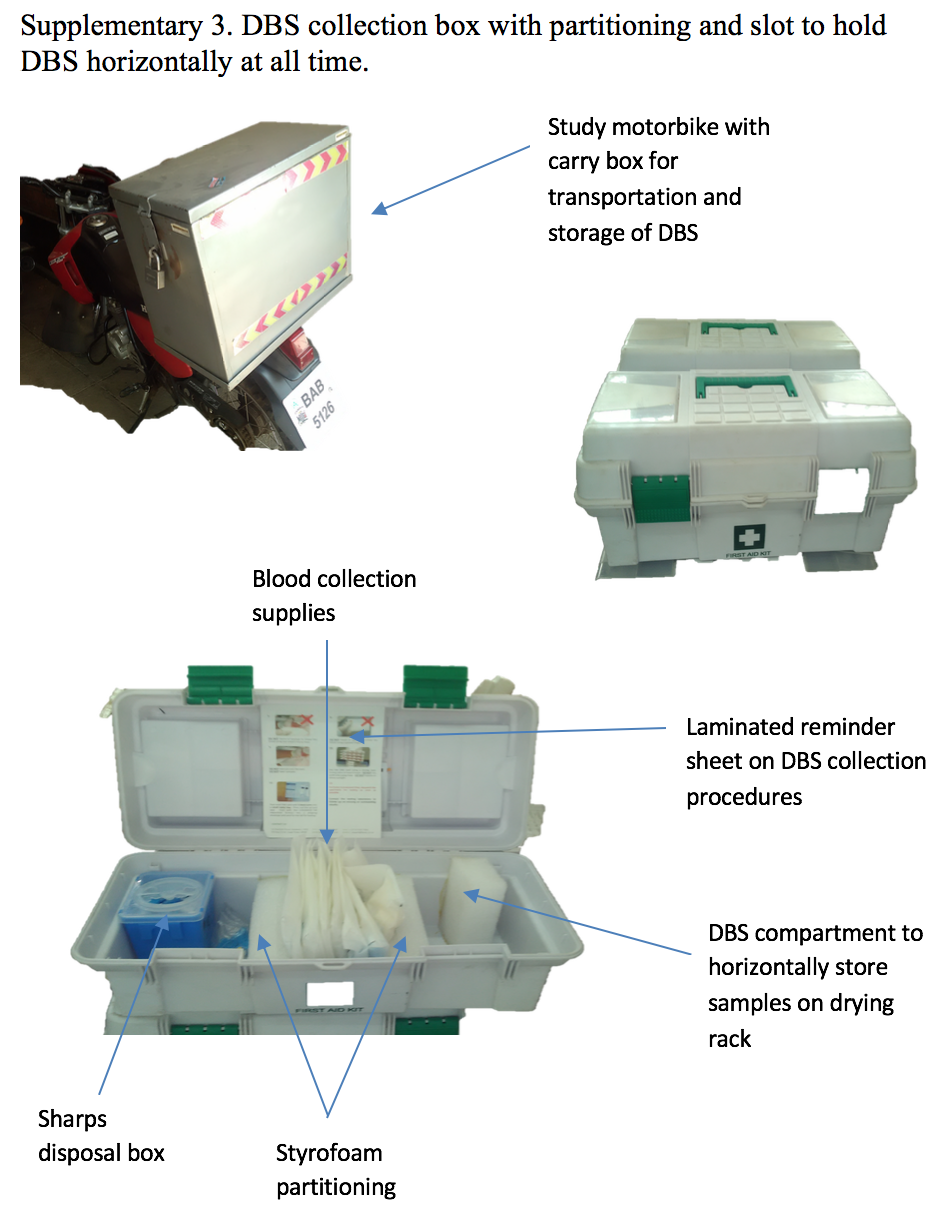

Supplement: S3 Appendix — Modified first aid box partitioned with Styrofoam to hold dired blood spots (DBS) horizontally at all times during transportation. (TIFF) [file pone.0223573.s003.tiff]
